# Bidirectional Angle-Tolerant Polarization-Tuned Filtering and Wide-Range Refractive Index Sensing Based on Metal Film Coated Nanograting

**DOI:** 10.3390/nano11010047

**Published:** 2020-12-27

**Authors:** Wenli Cui, Qiannan Wu, Bo Chen, Xufeng Li, Xiaolin Luo, Wei Peng

**Affiliations:** 1 College of Science, North University of China, Taiyuan 030051, China; qiannanwoo@nuc.edu.cn (Q.W.); bochen@nuc.edu.cn (B.C.); 2 School of Applied Science, Taiyuan University of Science and Technology, Taiyuan 030024, China; xfli@tyust.edu.cn; 3 College of mechatronic Engineering, North University of China, Taiyuan 030051, China; ouwenlxl@163.com; 4 School of Physics, Dalian University of Technology, Dalian 116024, China; wpeng@dlut.edu.cn

**Keywords:** bidirectional filtering, bidirectional RI sensing, SPP resonance, dipole resonance, nanograting-coated single-layer metal film

## Abstract

The miniaturization and integration of photonic devices are new requirements in the fast-growing optics field. In this paper, we focus on a feature-rich sub-wavelength nanograting-coated single-layer metal film. The numerical results show that the reflection behaviors of this proposed structure can realize bidirectional dual-channel ultra-narrowband polarized filtering and bidirectional wavelength-modulated sensing in a wide refractive index (RI) range from 1.0 to 1.4 for incident angle of 10° with transverse-magnetic (TM) polarized illumination at wavelengths between 550 nm to 1500 nm. Moreover, the bidirectional properties of filtering and sensing are not obviously decreased when increasing incident angle from 10° to 30°, and decreasing incident angle from 10° to 0°. The calculated RI sensitivity can be up to 592 nm/RIU with a high figure of merit (FOM) of 179.4 RIU^−1^. More to the point, this nanograting has a simple structure and is less sensitive to the height and shape of grating ridge, which provides great convenience for the fabrication of devices. The other thing that is going on is that this structure can also realize synchronously tunable color filtering, including green to red, with high color purity in the visible band by choosing the period. The underlying physical mechanism is analyzed in detail, and is primarily attributed to surface plasmon polariton (SPP) resonance and dipole resonance at double plasmon resonance wavelengths. This work has tremendous potential in developing multipurpose and high-performance integrated optical devices such as spectral filters, colored displays and plasmon biomedical sensors.

## 1. Introduction

In recent years, the various plasmonic nanostructures have drawn great attention in applications such as thermal emitters [1], color filters [2,3,4], solar cells [5,6], chemical and biological sensors [7,8,9], imaging devices [10,11,12], etc. Among these nanostructures, periodic nanogratings are playing a crucial role in this rapidly growing plasmonics research field [13,14,15]. A series of nanogratings with different structural configurations have been designed and discussed. For instance, a sandwiched two-layer grating polarizer with high extinction ratio and wide bandwidth was presented for its moderate fabrication tolerance [16]. A high-performance plasmonic transmission structure consisting of two cascaded ultrathin metallic nanogratings was proposed, which obtained near-perfect transmission and ultra-narrow bandwidth of 20 nm in sensing and filtering applications [17]. A palladium-coated narrow groove nanograting was designed, which was able to achieve highly sensitive hydrogen sensing capacity at visible and near-infrared wavelengths in the case of normal or angular illumination from the side of narrow groove [18]. Despite there being a variety of nanogratings described in previous reports, most studies have focused mainly on only one or two typical optical characteristics. The nanogratings with more typical optical properties or multi-purpose integration are usually lacking and limited.

In this paper, we present a feature-rich sub-wavelength nanograting-coated single-layer metal film. It can perform well in bidirectional ultra-narrowband polarized filtering, wide-range RI sensing for wavelength modulation with TM polarized illumination at incident angle of 10°. More importantly, these performances exhibit good angular tolerances when increasing the incident angle from 10° to 30°, or decreasing incident angle from 10° until 0°. Furthermore, in particular, our designed structure is less sensitive to the height and shape of the grating ridge, which is highly favorable to the fabrication of devices. It is particularly worth mentioning here that the tunability of color filtering including green to red with a high color purity in the visible region can be also realized synchronously by tuning the period of nanograting. To trace its physical origin, SPP resonance and dipole resonance play key roles. This structure is promising for multifunctional integrated applications in multispectral filtering, colored displays and plasmon biomedical sensing.

## 2. Structure and Method

**Figure 1:**
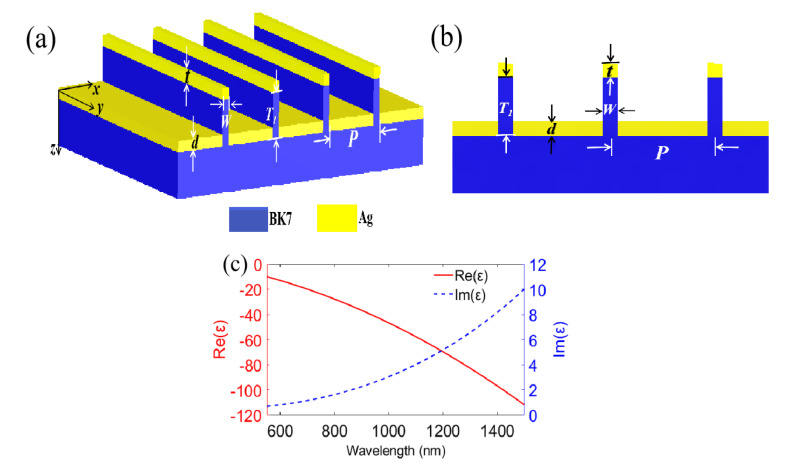
Nanograting-coated single layer metal film and material parameters. (**a**) Three-dimensional schematic diagram; (**b**) Cross-section of designed grating; (**c**) The relative permittivity of silver.

Figure 1a,b illustrate the schematics of this bidirectional multi-functional nanograting, which is coated with a thin Ag film. The geometric parameters of the proposed structure include period (*P*), grating width (*W*) and height (*T*_1_). *d* is defined as the thickness of the bottom metal layer, *t* is defined as the thickness of the top metal layer. If the two thicknesses are equal, *d* and *t* can be identically expressed as *a*. Figure 1c gives the relative permittivity of Ag material in the discussed wavelength range from 550 to 1500 nm. Its real part is negative (solid red line) and meets the excited requirement for surface plasmon wave on the metal/dielectric interfaces. Its imaginary part (dashed blue line) is relatively smaller, and indicates lower material loss for the proposed waveband. This structure can be fabricated by using e-beam lithography and lift-off process. In addition, its optical characteristics are analyzed by using commercial software COMSOL MULTIPHYSICS, which is based on the finite element method. Here, we employ a wave optics module and a frequency domain interface in order to solve the time-harmonic electromagnetic field distributions. The permittivity of Ag, as noted above, is described by the Drude–Lorentz model, which is given by [19]

(1) $$\varepsilon_{m}(\omega )=\varepsilon_{r}-\frac{\omega_{p0}^{2}}{\omega (\omega +i\gamma_{0})}-\frac{\Delta \varepsilon_{0}\Omega_{0}^{2}}{\omega^{2}-\Omega_{0}^{2}+i\omega \Gamma_{0}}$$

In Equation (1), the first two items are given by the Drude model, where ω is the angle frequency, $\omega_{p_{0}}$ is the plasma frequency, and $\gamma_{0}$ is the damping coefficient. The third term is the Lorentzian term, where $\Omega_{0}$ and $\Gamma_{0}$ stand for, respectively, the oscillator strength and spectral width of the Lorentz oscillators, and ${\Delta \varepsilon }_{0}$ can be interpreted as a weighting factor. Here, we use the values of the Drude–Lorentz model as follows: $\varepsilon_{r}=4.6$, $\omega_{p_{0}}=1.37\times 10^{16}\mathrm{rad}/s$, $\gamma_{0}=1.06\times 10^{14}\mathrm{rad}/s$, $\Delta \varepsilon_{0}=1.10$, $\Omega_{0}=7.43\times 10^{15}\mathrm{rad}/s$, $\Gamma_{0}=1.82\times 10^{15}\mathrm{rad}/s$. The RI of BK7 glass material is a constant of 1.513. The above-mentioned parameters and Equation (1) are all set in global definitions of the model builder for employing the software. A plane wave illuminates bidirectionally from the bottom or the top of this nanograting, whose electric field component paralleling to the *x* or *y* axis indicates a TM or transverse electric (TE) polarization. To achieve high precision, reduce processing time, and analyze mechanism more easily, we choose one or two unit cells of nanograting in the following calculation. The periodic boundary conditions are set in the x direction, and perfectly matched layers are used in the ±z direction. The characteristic spectra are achieved by numerical calculation with 2 nm resolution in the wavelength range from 550 to 1550 nm. All simulation results are normalized to the incident light power.

## 3. Results and Discussion

### 3.1. Description of Spectral Characteristics

**Figure 2:**
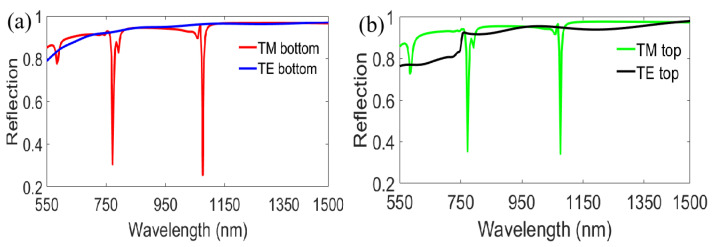
Reflection spectra of nanograting-coated single-layer metal film at an incident angle of 10° for TM and TE polarization: (**a**) Incidence from the bottom; and (**b**) Incidence from the top.

**Figure 3:**
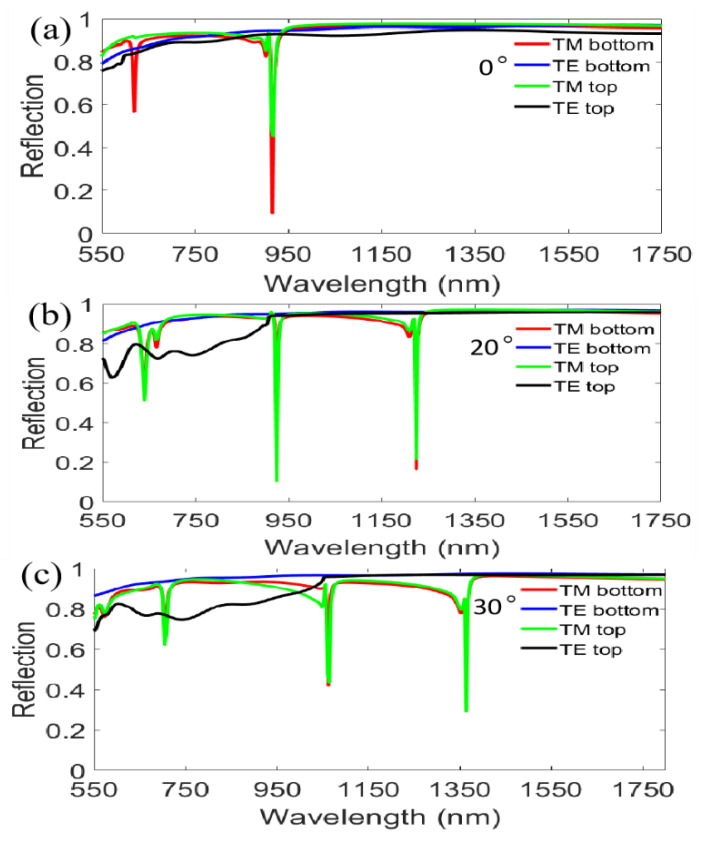
Reflection spectra of the designed nanograting with TM and TE polarized illuminations from the bottom and the top under different incident angles of (**a**) 0°; (**b**) 20° and (**c**) 30°.

First, we investigated the reflection spectra of nanograting with single layer metal film at 10° incidence from the bottom or the top with optimized structure parameters *P* = 600 nm, *W* = 30 nm, *T*_1_ = 180 nm, *d* = *t* = *a* = 40 nm. As shown in Figure 2a, there are two obvious ultra-narrowband reflection dips at λ=772 nm and λ=1076 nm, respectively, under TM polarized incidence for the bottom illumination. The narrowest full width at half maximum (FWHM) of the two resonant dips is predicted to be excellent, at 6 nm. In Figure 2b, it is also clearly seen that two obvious ultra-narrowband reflection dips are positioned at the same wavelengths of λ=772 nm and λ=1076 nm, respectively, under TM polarized incidence for the top illumination. As a comparison, the reflection spectra for TE polarized incidence are also plotted in Figure 2a,b (blue line and black line, respectively). The simulated results show that our designed structure has the characteristics of bidirectional dual-channel ultra-narrowband polarized filtering and high reflection of bidirectional no-polarized selectivity at wavelengths between 550 nm to 1500 nm. Subsequently, we also investigated the reflection spectra of single layer metal film grating when reducing the incident angle from 10° to 0° and increasing the incident angle from 10° to 30° in 10° steps, as depicted in Figure 3. Here, we observe that the characteristic of dual-channel ultra-narrowband polarized filtering is changed only when the incident angle is reduced to 0°. Namely, the reflection resonant dip at the short wavelength disappears with 0° incidence when adjusting the incident direction from the bottom to the top for TM polarization, as shown in Figure 3a. However, it is worth noting that the characteristic of bidirectional dual-channel ultra-narrowband polarized filtering can still be well kept at some other angles such as 20° and 30°, as shown in Figure 3b,c. Additionally, the highly reflected characteristic of bidirectional no-polarized selectivity in near infrared region is similarly compatible with incident angle whether decreasing *θ* from 10° to 0°, or increasing *θ* from 10° to 30°.

**Figure 4:**
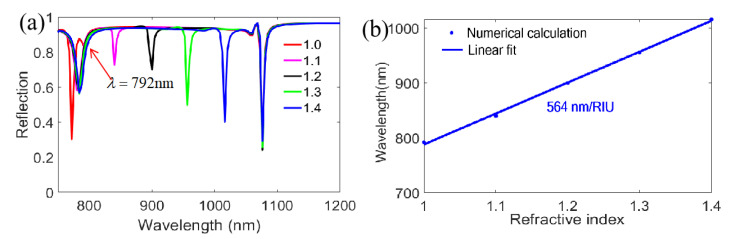
Investigation of sensing performance for TM polarization on the bottom with 10° incident angle. (**a**) Reflection spectra of different RIs; (**b**) Linear response of the resonant wavelength for the little reflection dip (at λ=792 nm in air).

Considering the wavelength range of 550~1500 nm is interesting for developing surface plasmon sensors [20]; consequently, the sensing prospects in a wide RI region from 1.0 to 1.4 for TM polarized illumination on the bottom with incident angle $\theta =10^{{}^{\circ}}$ are calculated in Figure 4. Clearly, we find that when the RI surrounding nanograting ridges is changed between 1.0 and 1.4, the RI responses of the first reflection resonant dip (at λ=772 nm in air) and the second reflection resonant dip (at λ=1076 nm in air) for wavelength are both extremely slight compared to the little reflection resonant dip (at λ=792 nm in air) between them, as revealed in Figure 4a. A clearer detail is exposed in Figure 4b, where a good linear relation between the RI and resonant wavelength for the little reflected dip is achieved, and a sensitivity (S=Δλ/Δn) of 564 nm/RIU can be obtained.

**Figure 5:**
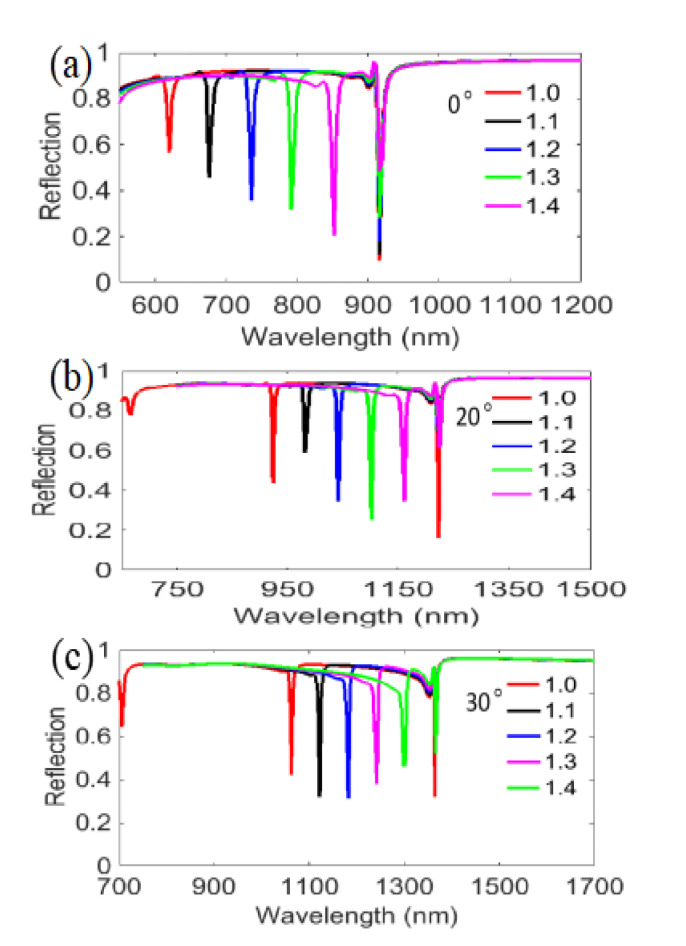
Reflection spectra for TM polarized illumination on the bottom with different RIs at discussed incident angles of (**a**) 0°, (**b**) 20° and (**c**) 30°.

**Figure 6:**
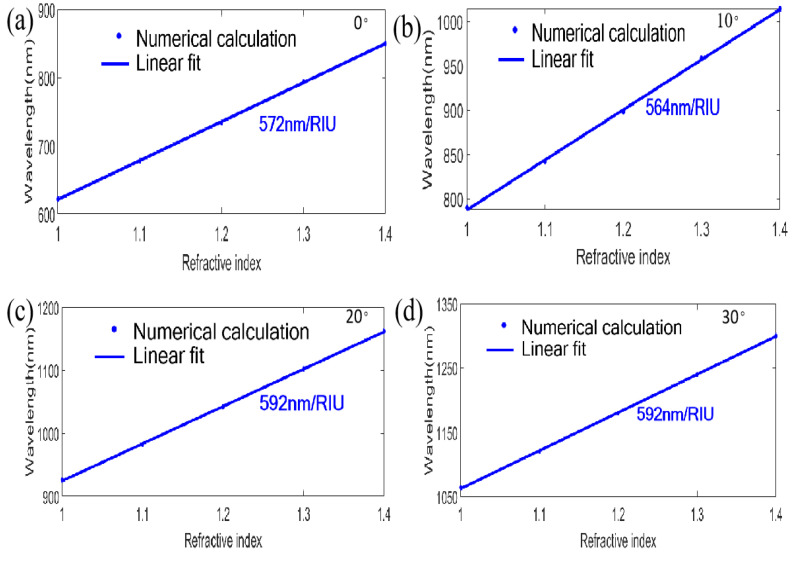

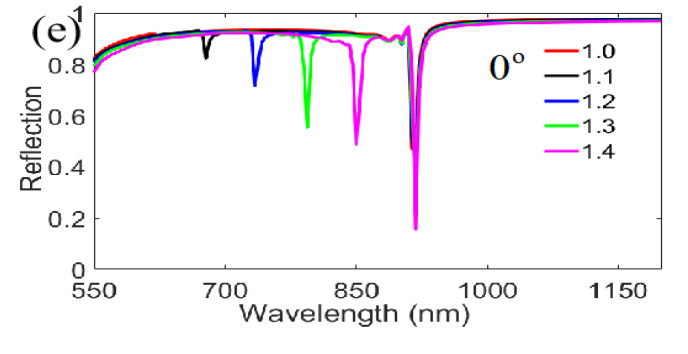
Investigation of sensing performance for TM polarized incidence on the top with different RIs at $\theta =0^{{}^{\circ}}\sim 30^{{}^{\circ}}$. Linear response of the resonant wavelength for (**a**) 0°, (**b**) 10°, (**c**) 20° and (**d**) 30°; (**e**) Reflection spectra with different RIs at $\theta =0^{{}^{\circ}}$.

Then, we investigated the influences of different incident angles for TM polarized illumination on the bottom of the designed nanograting, as shown in Figure 5. Above all, in Figure 5a, we observe that the first reflection resonant dip (at λ=620 nm in air) exhibits an evident redshift for $\theta =0^{{}^{\circ}}$ incidence in the whole wide RI region from 1.0 to 1.4, which is obviously different from the first reflection resonant dip (at λ=772 nm in air) of $\theta =10^{{}^{\circ}}$ (see Figure 4a), namely, the first reflection resonant dip represents a high wavelength sensibility when tuning the incident angle to $\theta =0^{{}^{\circ}}$. Meanwhile, Figure 5b,c also represent similar features when increasing the incident angle to $20^{{}^{\circ}}$ and $30^{{}^{\circ}}$. Moreover, through further calculation, we find that a higher RI sensitivity of 580 nm/RIU can be achieved at $\theta =0^{{}^{\circ}}$ compared to $\theta =10^{{}^{\circ}}$ for the little resonant dip (at λ=792 nm in air). The further calculation indicates that the FOM (FOM= S/FWHM) is also better, with a value of 96.7 RIU^−1^, compared to 94.0 RIU^−1^ for $\theta =10^{{}^{\circ}}$. Additionally, the RI sensitivities and the FOM for $\theta =20^{{}^{\circ}}$ and $\theta =30^{{}^{\circ}}$ are also considered, which are 592 nm/RIU and 179.4 RIU^−1^, and 590 nm/RIU and 140.5 RIU^−1^ at two different incident angles. Considering the simulated results for evaluating sensing performance in Figure 5 based on the bottom incidence for TM polarization, we also calculate the results of the top incidence at $\theta =0^{{}^{\circ}}\sim 30^{{}^{\circ}}$ for TM polarization. As shown in Figure 6a–d, there are good linear relationships between the RI of the medium and the resonance wavelength. The RI sensitivities are as follows: 572 nm/RIU for $\theta =0^{{}^{\circ}}$, 564 nm/RIU for $\theta =10^{{}^{\circ}}$, 592 nm/RIU for $\theta =20^{{}^{\circ}}$, 592 nm/RIU for $\theta =30^{{}^{\circ}}$, which are basically consistent with the data of the bottom incidence, as mentioned above. Additionally, it is particularly pointed out that although the first reflection resonant dip (at λ=620 nm in air) under normal incidence from the bottom with TM polarization disappears when adjusting the incident direction to the top (see Figure 3a), the reflection spectra can still exhibit the peculiarity of dual-channel filtering and sensing function, with an increase in the RI from 1.0 to 1.4 (see Figure 6e).

In addition, to compare the results obtained here with other research results in this field, the performances of some relevant raster-based systems are shown in Table 1. These data include those from narrow groove nanograting, SPP sensing nanograting, PMMA-protected Ag nanograting and nanograting-based Kretschmann configurations. By contrast, we can find that the metal film-coated nanograting has an excellent FOM and a wider RI detecting range, whose comprehensive sensing performances are better than the other configurations.

In conclusion, we conclude that our designed structure possesses bidirectional angle-tolerant polarization-tuned filtering in a range of $0^{{}^{\circ}}\sim 30^{{}^{\circ}}$ and wide-range RI sensing in a range of 1.0~1.4 in the visible-near infrared regions of 550 nm~1500 nm.

### 3.2. Investigation of Physical Mechanism

**Figure 7:**
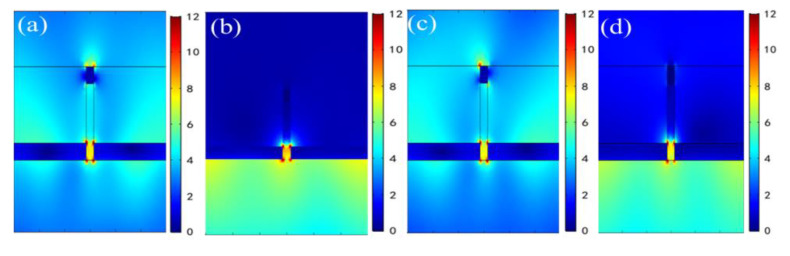
Electric field distributions of two different reflection resonant dips at $\theta =10^{{}^{\circ}}$ for TM polarization from the bottom and the top. (**a**) λ=772 nm on the bottom; (**b**) λ=1076 nm on the bottom; (**c**) λ=772 nm on the top; (**d**) λ=1076 nm on the top.

**Figure 8:**
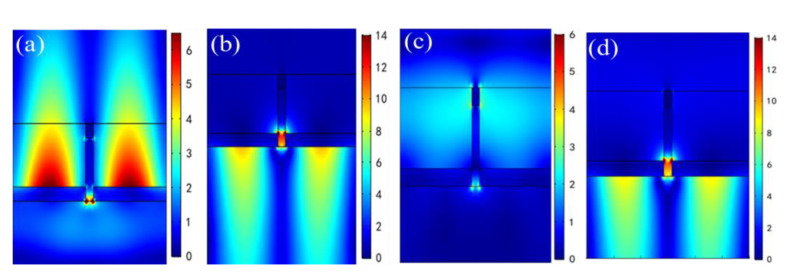
Electric field distributions of two different reflection resonant dips at $\theta =0^{{}^{\circ}}$ for TM polarization from the bottom and the top. (**a**) λ=620 nm on the bottom; (**b**) λ=916 nm on the bottom; (**c**) λ=620 nm on the top; (**d**) λ=916 nm on the top.

To further understand the physical origin of the extraordinary optical characteristics in the wavelength region of 550~1550 nm for this metal film-coated nanograting, more detailed results are provided in subsequent Figures. Firstly, Figure 7 illustrates the normalized electric field distributions of the two reflected resonant dips at λ = 772 nm and λ = 1076 nm under θ=$10^{{}^{\circ}}$ incidence with TM polarized illumination from the bottom and the top of the structure, respectively. It can be clearly seen that the influence of adjusting incident light direction is extremely slight for the spatial distributions of electric fields at both plasmon resonant dips. Moreover, the electric dipolar resonance modes are mainly excited at dual resonant wavelengths. Figure 8 describes normalized electric field distributions for a reduced incident angle of θ = $0^{{}^{\circ}}$. It can be clearly observed that there are remarkable differences between the two sets of Figures: On the one hand, as the dual resonant dips are blue shifted to λ=620 nm and λ=916 nm, respectively, for the bottom incidence, the spatial electric field distributions represent apparent characteristics of SPP at metal/dielectric interfaces. At the wavelength of λ=620 nm, the strong electric field is confined to the upper surface of the bottom Ag film and has its maximum on the surface of the Ag film/air layer; at the wavelength of λ=916 nm, the strong electric field is localized to the lower surface of the bottom Ag film and has its maximum on the surface of Ag film/BK7 substrate. On the other hand, when adjusting the incident direction of TM polarized light from the bottom to the top, although it does not cause apparent changes of the field distributions for λ=916 nm, the reflected dip of λ=620 nm disappears in the case of adjusting the direction of incident light (see Figure 3a), and the corresponding field distribution also indicates that the stimulation of SPP is non-existent (see Figure 8c). Therefore, we draw a conclusion that the remarkable RI response of the first reflected resonant dip for wavelength under normal incidence with TM polarization from the bottom is mainly attributed to the excitation of SPP resonance on the upper surface of bottom Ag film for the designed nanograting. This can be further verified by the SPP excitation condition on the Ag film/air interfaces, as described in [24]:

(2) $$n\frac{2\pi }{\lambda }\sin\theta -N\frac{2\pi }{P}=-\frac{2\pi }{\lambda }\sqrt{\frac{\varepsilon_{m}(\omega )\varepsilon_{d}}{\varepsilon_{m}(\omega )+\varepsilon_{d}}}=k_{spp}\;N=0,\pm 1,\pm 2,\cdot \cdot \cdot$$

where λ is incident light wavelength, *P* is the grating period, *N* is diffraction order, *θ* is incident angle, *n* is the RI, $\varepsilon_{m}$ and $\varepsilon_{d}$ represent relative permitivities of metal and dielectric, respectively. According to Equation (2), we can calculate that the theoretical wavelength of the diffraction order *N* = 1 is 622 nm, and this agrees well with the numerical wavelength of 620 nm.

**Figure 9:**
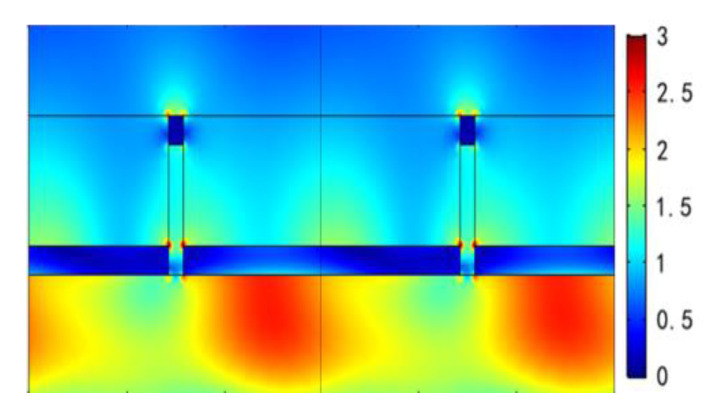
Electric field distribution of the little dip (λ = 792 nm, *n* = 1.0) between the first dip and the second dip at *θ* = 10° for TM polarized incidence from the bottom of single layer metal film grating.

Similarly, the RI response of the little dip between the first dip and the second dip at *θ* = 10° for TM polarization on the bottom incidence can also be understood with the excitation of SPP resonance mode, as shown in Figure 9, which is also located on the lower Ag film/air interfaces.

**Figure 10:**
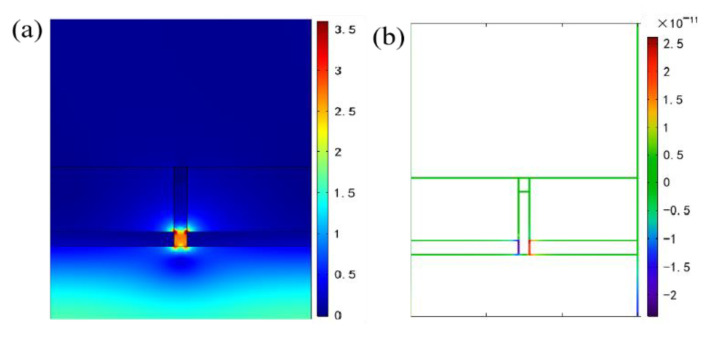
Electric field and charge distributions in the near infrared region at *θ* = 10° with TM polarized incidence from the bottom of single layer Ag film grating. (**a**) Normalized spatial electric field distribution at a typical resonant wavelength of λ= 1450 nm; (**b**) The corresponding charge distribution at λ= 1450 nm.

However, what is different from Figure 8a is that the excitation intensity of the SPP on the lower Ag film/air interface is relatively weaker. This further confirms the fact that the highest RI sensitivity for wavelength modulation can be achieved at θ = 0° (*S* = 580 nm/RIU), rather than at θ = 10° (*S* = 564 nm/RIU), as mentioned earlier. Additionally, Figure 10 gives the distributions of spatial electric field and charge at θ = 10° with TM bottom incidence for high reflected characteristic of bidirectional no-polarization selectivity in near infrared waveband. At a typical resonant wavelength of 1450 nm, we observe that positive and negative oscillating charges are markedly stimulated at the right end of the left Ag film and the left end of the right Ag film near the substrate as depicted in Figure 10b. Thereby, we conclude that the formation mechanism of high reflected feature for discussed structure is closely related to the existence of electric dipolar resonance mode.

**Figure 11:**
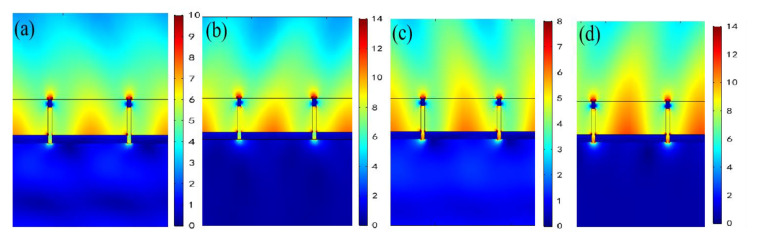
Electric field distributions of the first reflection resonant dip for TM polarized incidence from the bottom and the top at *θ* = 20° and *θ* = 30°. (**a**) = 924 nm on the bottom for *θ* = 20°; (**b**) λ = 924 nm on the top for *θ* = 20°; (**c**) λ = 1062 nm on the bottom for *θ* = 30°; (**d**) λ = 1062 nm on the top for *θ* = 30°.

Based on the above analysis of θ = 0° and θ = 10°, we go on to pay attention to the field distributions of the first reflection resonant dip at θ = 20° and θ = 30° as described in Figure 11. Clearly, we see that there are SPP resonance modes that are excited on the lower Ag film/air interfaces for the first reflection resonant dip under TM incidence, whether the bottom illumination or the top illumination, whether θ = 20° or θ = 30°. These results indicate that Figure 11 is extremely helpful for explaining the sensing mechanism of wavelength modulation at the first reflected resonant dip for Figure 5b,c. Additionally, the excitation intensities of the SPP in Figure 11 are relatively stronger than those in Figure 9, which is in good agreement with the fact that higher RI sensitivities can be obtained at θ = 20° and θ = 30° than at θ = 10°, as noted above.

### 3.3. Influence of Geometrical Parameters

**Figure 12:**
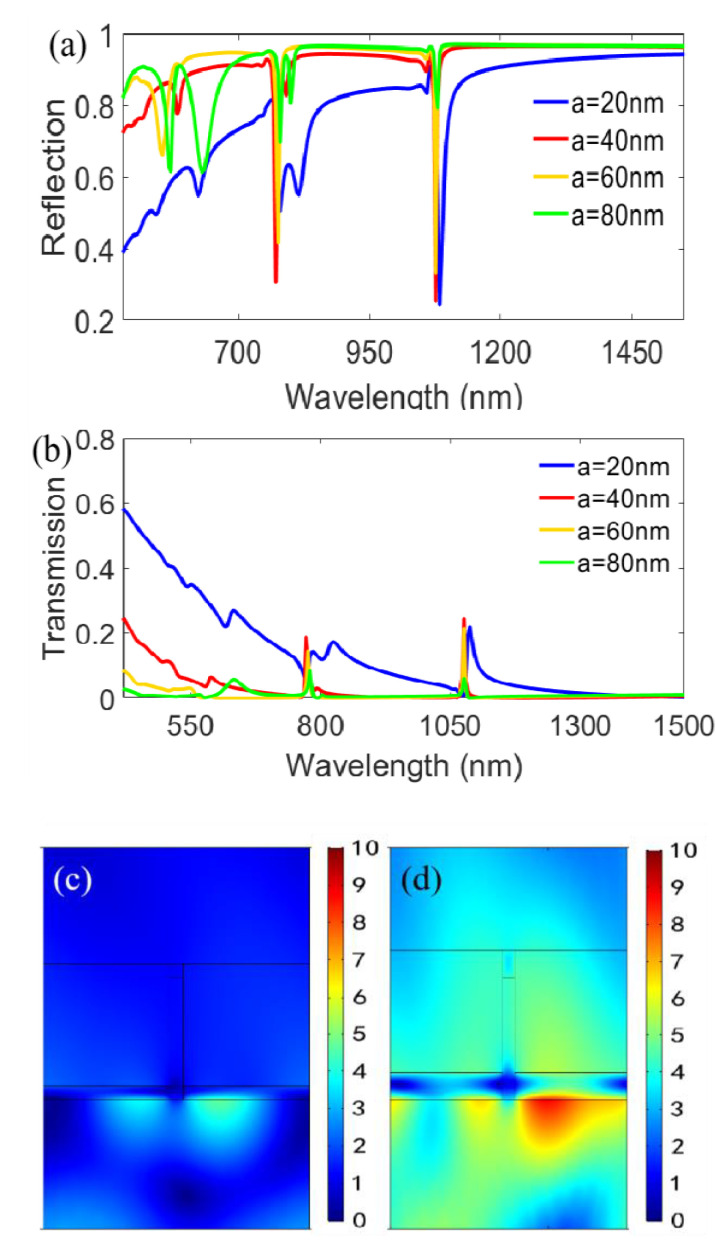
Influences of the metal film thickness with synchronous change for the top and bottom under TM polarized incidence at *θ* = 10° from the structural bottom. (**a**) The calculated reflection spectra at different Ag film thicknesses; (**b**) The calculated transmission spectra at different Ag film thicknesses; Magnetic field distributions of (**c**) *a* = 20 nm and (**d**) *a* = 40 nm for the first reflection dip.

**Figure 13:**
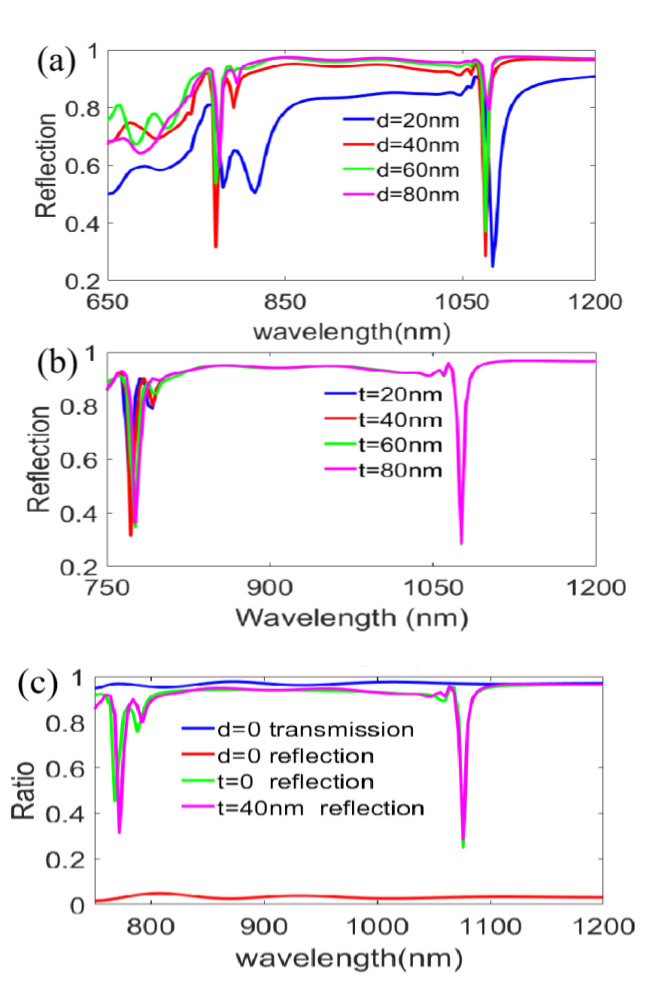
Influence of the metal film thickness with non-synchronous change of the top and bottom on the characteristics for TM polarized incidence at *θ* = 10° from the structural bottom. (**a**) Change thickness of the bottom film; (**b**) Change thickness of the top film; (**c**) Spectral comparisons with different metal film thicknesses.

We investigate the influences of structural parameters for the reflected spectra under TM polarized incidence with *θ* = 10° from the bottom of the designed structure. As shown in Figure 12a,b, when increasing the thicknesses of the top and the bottom metal film from 20 to 80 nm in 20 nm steps synchronously, the reflectivities of the double plasmon resonant dips increase; meanwhile, the transmittances decrease as a whole, but *a* = 20 nm to *a* = 40 nm is an exception, especially for the first reflection dip. To analyze the details, we give the magnetic field distributions of *a* = 20 nm and *a* = 40 nm in Figure 12c,d. By contrast, we find that the characteristic of lower reflectivity or higher transmittance at *a* = 40 nm is related to stronger localized field on the lower Ag film/air interfaces and the lower Ag film/BK7 interfaces. Meanwhile, the penetration depths of magnetic field show a breakover behavior within the bottom Ag film and result in a higher transmittance and a lower reflectivity. Figure 13 further gives the influences of asynchronous modulation of Ag film thickness for the top and the bottom. When increasing of the bottom film thickness (*d*) from 20 to 80 nm in Figure 13a, in general, the reflectivities of the reflection spectra for the double resonant dips are rising, except *d* = 40 nm, similarly to Figure 12a. However, the reflectivities of reflected spectra are insensitive to the change of the top film thickness (*t*), as revealed in Figure 13b. As a result, we conclude that the optimization of the bottom film thickness is crucial to the formation of spectral characteristics. Figure 13c can further prove this point. When removing the bottom film (*d* = 0), the reflectivity is rapidly reduced and the transmittance is evidently increased, but when removing the top metal film (*t* = 0), the variation in the reflection spectra is negligible.

**Figure 14:**
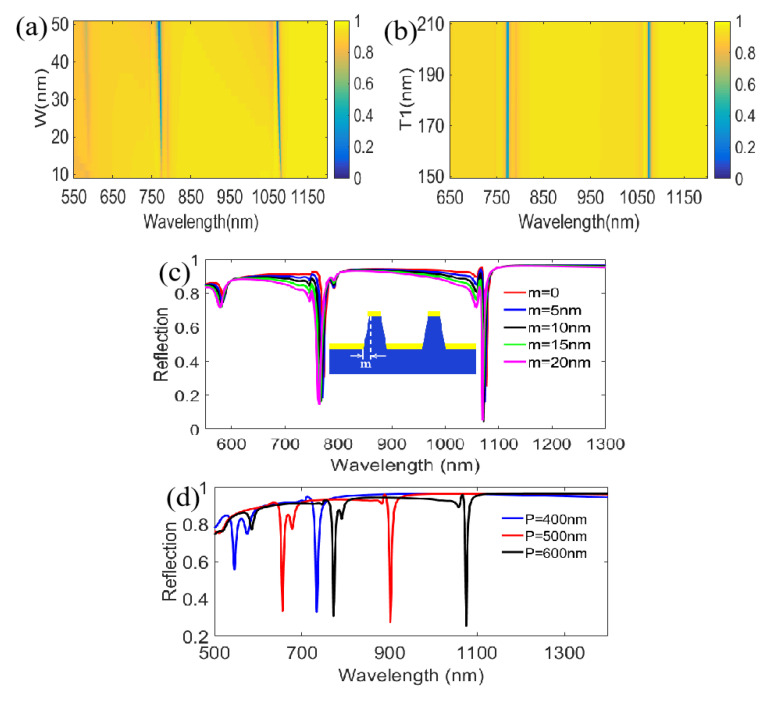
Influences of different parameters on the reflection spectra for TM polarized incidence at *θ* = 10° from the structural bottom. (**a**) Change of the grating width; (**b**) Change of the grating height; (**c**) Change of grating ridge shape; (**d**) Change of the grating period.

Then, the influences of other structural parameters including width, height and period were also investigated under TM polarized incidence at *θ* = 10° from the structural bottom for the reflection spectra. Figure 14a firstly shows the calculated results of changing grating width (*W*). As W increases from 10 nm to 50 nm, the electric dipolar resonance, which is presented at the right end of the left Ag film and at the left end of the right Ag film near the substrate, is weakened, as shown in Figure 7a,b. Accordingly, slight blue-shifts appear at the double resonant dips. Meanwhile, the reflectivities are also relatively decreased. Figure 14b displays that the grating height (*T*_1_) is increased from 150 to 210 nm, but it is worth noting that there are no evident changes in the double resonant dips, which indicates that the electric dipolar resonance mentioned is unaffected in the case of changing *T*_1_. Subsequently, Figure 14c gives the influences of different grating ridge shape for the reflection spectra. Interestingly, the results of trapezoidal shape are close to the results of rectangular shape, which is highly convenient for the fabrication of devices. Figure 14d depicts the reflection characteristics with varying period (*P*). With an increase in *P* from 400 to 600 nm, the double resonant dips exhibit different red-shifts, indicating the function of tunable filtering. In particular, for the first resonant dip, there are wavelength shifts from 546 nm to 772 nm, which indicate that a tunable color filtering from green to red with high color purity can be realized in the visible band (here, the minimum of narrow linewidth is 4 nm at λ=656 nm for *P* = 500 nm and λ=772 nm for *P* = 600 nm, respectively) as shown in Figure 14d.

## 4. Conclusions

In summary, we numerically illuminated an Ag-coated nanograting with great optical properties, including bidirectional dual-channel ultra-narrowband polarized filtering, high reflection of bidirectional depolarization, and bidirectional wavelength-modulated sensing in a wide RI range of 1.0~1.4 at 10° incidence from 550 to 1500 nm. More notably, the above properties have excellent tolerances with respect to incident angle when increasing *θ* from 10° to 30° and decreasing *θ* from 10° to 0°. The calculated results reveal that the proposed structure provides a maximum RI sensitivity of up to 592 nm/RIU, with a FOM of 179.4RIU^−1^, at 20° incidence. Moreover, this structure can also realize tunable color filtering, including green to red, with high color purity in the visible band by tuning the period. The underlying physical mechanism is analyzed in detail, and is mainly associated with the excitations of SPP resonance and dipole resonance at dual plasmon resonant dips. This work provides a new pathway for the design and development of multiple function integrated optical devices such as multispectral tunable filter, colored displays and plasmon biomedical sensor.

## Figures and Tables

**Table 1:** Comparison of sensing capabilities with earlier reported works based on nanograting.

Structure	Maximum S (nm/RIU)	RI Range	FOM (RIU^−1^)	Reference
Narrow groove nanograting	500	1.33~1.4	—	[20]
SPP sensing nanograting	440	1.2~1.5	60	[21]
PMMA-protected Ag grating	445	1.33~1.36	—	[22]
Nanograting-based	1100	1.33~1.36	20	[23]
Kretschmann configurations				
Metal film-coated Nanograting	592	1.0~1.4	179.4	this work
